# Current News

**Published:** 1898-09

**Authors:** 


### Current News.

### NEW JERSEY STATE DENTAL SOCIETY.

### FIRST DAY.

The Twenty-eighth Annual Session of the New Jersey State Dental Association commenced in the Auditorium, Asbury Park, N. J., July 21, 1898.

At the opening session the annual address of the president, J. L. Crater, of Orange, was delivered. He dwelt upon the work of the society during the past year, and reported that there had been no deaths. He also referred to the fact that the treasury was in a flourishing condition.

The roll was called and several names were proposed for membership. Reports were also received at the morning session from the National Museum at Washington and the National Dental Association.

At the afternoon session the following essays were read: "A New Sectional Block Tooth," by Allison R. Lawshe, D.D.S., of Trenton; "Practical Experience with a few Homœopathic Remedies in Dental Practice," by William J. Wallace, M.D., of Glen Falls, N. Y.; "An Effective Method of treating Chronic Alveolar Abscess and Molars having Exposed Pulps Difficult to extirpate," by Frank G. Gregory, D.D.S., of Newark.

Two essays of considerable interest occupied the time of the evening session. The subject of the first was: "A Study of the Physiological and Pathological Conditions of the Apical Portion of the Cementum," by I. P. Wilson, D.D.S., of Burlington, Iowa.

This was followed by J. A. Waas, of Hammonton, N. J., who read an essay on "My Experience with Nerve Mummification."

### SECOND DAY.

The second day's sessions were held in the Auditorium. President J. L. Crater, of Orange, was in the chair, and the roll call in the morning showed about one hundred and fifty delegates present.

The morning session was devoted to the reading and discussion of several papers.

At this session the following papers were read: "Alveolar Abscess and Caries of the Maxilla," by W. G. Chase, D.D.S., Philadelphia; "A Plea for the More Scientific and Careful Study of Materia Medica as a Branch of Dental Education," by Henry H. Merrell, Ph.D., M.D., Chicago; "Details of an Amalgam Filling, exhibiting a Regulating Appliance for regaining the Interproximate Space," by George W. Schwartz, M.D., D.D.S., Chicago; "The Evolution of Dental Materia Medica," by William H. Trueman, D.D.S., Philadelphia.

The afternoon session was devoted to clinics.

During the afternoon several interesting clinics were performed in the presence of a large gathering of dentists from all parts of the State. Dr. A. Irwin, Camden, "A Case in Orthodontia;" Dr. C. LeRoy, New York, "Filling with the New S. S. W. Moss Gold;" Dr. D. C. Baker, New York, "Movable Bridge-Work and Crown-Work;" Dr. H. C. Register, Philadelphia, "The Register Method of Operating;" D. Ashley Faught, Philadelphia, "A New Process of Practical Work;" Dr. Charles A. Meeker, Newark, N. J.," Bleaching a Tooth by Cataphoresis."

THIRD DAY.

The election of officers for the ensuing year was the principal business. The election resulted as follows:

President, Dr. J. Allen Osmun, Newark; Vice-President, W. E. Truex, Freehold; Secretary, C. A. Meeker, Newark; Treasurer, H. A. Hull, New Brunswick.

*Executive Committee.*—Oscar Adelberg, Elizabeth; F. E. Riley, Newark; F. G. Gregory, Newark; R. M. Sanger, East Orange.

*Membership Committee.*—F. L. Hindle, New Brunswick; F. L. Fish, Newark; C. S. Hardy, Summit; C. W. Hoblitzell, Jersey City; W. H. Pruden, Paterson.

Dr. J. C. Barlow, of Jersey City, was recommended to the governor as a proper man for reappointment as a member of the State Board of Examination and Registration in Dentistry. Dr. L. Ashley Faught, of Philadelphia, was elected an honorary member of the Society.

During the morning a paper on "Dental Histology" was read by H. C. Register, D.D.S., of Philadelphia.

Before adjournment the following new members were elected: J. Starr Dunning, of Paterson; R. W. Emerson, of Plainfield; A. R. Lawshe, of Trenton; R. W. Jewett, of Keyport; Theodore Van Syckle, of Princeton; J. W. Fisher, of East Orange; and C. W. Hayden, Jr., of Hackensack.

The chairmen of the various committees all rendered detailed reports of the work under their provision. These reports were all received and filed, and will appear in the official printed proceedings of the sessions.

---

### SOUTHERN BRANCH OF THE NATIONAL DENTAL ASSOCIATION, SECOND ANNUAL MEETING, NEW ORLEANS, LA., FEBRUARY 9, 10, 11, AND 13, 1899.

LIST of committees for the joint meeting of the Southern Branch of the National Dental Association and the Louisiana State Dental Society:

No. 1. Committee of Arrangements, in subdivisions: Transportation Committee, Dr. Joseph Bauer (S.B.N.D.A.), chairman, New Orleans, La. Hotels and Quarters Committee, Dr. J. Rollo Knapp (S.B.N.D.A.), chairman, New Orleans, La. Hall, Exhibits, and Arrangements Committee, Dr. L. D. Archinard (L.S.D.S.), chairman, New Orleans, La.

No. 2. Committee on Clinics, Dr. T. P. Hinman (S.B.N.D.A.), chairman, Atlanta, Ga. Committee on Clinics (Louisiana State Dental Society), Dr. And. G. Friedrichs (L.S.D.S.), chairman, New Orleans, La.

No. 3. Committee on Publication, Dr. Shep. W. Foster (S.B.N.D.A.), chairman *ex officio*, Atlanta, Ga.

No. 4. Committee on Oral Hygiene, Dr. L. M. Cowardin, chairman, Richmond, Va.

No. 5. Committee on Prosthetic Dentistry, Dr. J. A. Dale, chairman, Nashville, Tenn.

No. 6. Committee on Orthodontia and Oral Surgery, Dr. Geo. Hardy, chairman, Baltimore, Md.

No. 7. Committee on Operative Dentistry, Dr. J. G. Fife, chairman, Dallas, Texas.

No. 8. Committee on Chemistry, Metallurgy, and Anatomy, Dr. Albert B. King, chairman, Baltimore, Md.

No. 9. Committee on Pathology, Materia Medica, and Therapeutics, Dr. H. H. Johnson, chairman, Macon, Ga.

No. 10. Committee on Microscopy, Histology, and Bacteriology, Dr. W. T. Martin, chairman, Yazoo City, Miss.

No. 11. Committee on Appliances and Improvements, Dr. T. M. Allen, chairman, Birmingham, Ala.

No. 12. Committee on Education, Literature, and Voluntary and Special Essays, Dr. T. P. Whitby, chairman, Selma, Ala.

Entertainment Committee, Dr. Joseph Bauer (L.S.D.S.), chairman, New Orleans, La.

Carnival Balls Invitations Committee, Dr. J. Rollo Knapp (S.B.N.D.A.), chairman, New Orleans, La.

C. L. ALEXANDER,
*Corresponding Secretary, Southern Branch National Dental Association.*

CHARLOTTE, N. C., July 12, 1898.

---

### SOUTHERN CALIFORNIA DENTAL ASSOCIATION.

A MEETING was held in Los Angeles, on June 23, by the dentists of Southern California for the purpose of organizing a dental society.

About fifty members of the profession were present, and organized the Southern California Dental Association, electing the following officers:

President, Dr. W. A. Smith, Los Angeles; First Vice-President, Dr. H. R. Harbison, San Diego; Second Vice-President, Dr. C. W. Sylvester, Riverside; Treasurer, Dr. J. M. White, Los Angeles; Secretary, Dr. L. E. Ford, 118½ South Spring Street, Los Angeles.

The society will hold its first regular meeting on September 2, 1898, in San Diego, Cal.

L. E. FORD, D.D.S.,
*Secretary.*

---

### MEETING OF THE VERMONT STATE BOARD OF DENTAL EXAMINERS.

THE next meeting of the Vermont State Board of Dental Examiners will be held at the Pavilion Hotel, Montpelier, October 18, 1898, 2.20 o'clock in the afternoon.

GEO. F. CHENEY,
*Secretary.*

ST. JOHNSBURY, VT.

